# COVID-19 and *Evolution, Medicine, and Public Health*

**DOI:** 10.1093/emph/eoad002

**Published:** 2023-01-28

**Authors:** Charles L Nunn

**Affiliations:** Department of Evolutionary Anthropology, Duke University, Durham, NC 27708, USA; Duke Global Health Institute, Duke University, Durham, NC 27708, USA; Triangle Center for Evolutionary Medicine (TriCEM), Duke University, Durham, NC 27708, USA

According to the World Health Organization, 6.7 million people have died from COVID-19 as of the start of 2023. These deaths are tragic with many societal ramifications. For example, more than 10 million children have lost caregivers globally through 1 May 2022 [1], while many others have suffered dramatic losses in educational attainment [2]. At times, the pandemic has overwhelmed healthcare services that have resulted in additional non-COVID death and suffering. COVID-19 has also caused sharp declines in mental health, particularly among children and adolescents [3, 4]. Mental disorders, such as depression and anxiety, are often debilitating and long-lasting, thus contributing greatly to years lived with disability. Some bright spots have also occurred, including the marked reduction in deaths due to influenza in the first year of the pandemic due to masking and social isolation and the rapid rollout of vaccines using new technologies such as mRNA vaccines, which offer great promise in battling other infectious diseases.

One lesson from the pandemic is the importance of interdisciplinary approaches for addressing complex problems. We cannot control a viral pandemic with just virology. We need epidemiologists, engineers, sociologists, political scientists, historians, medical doctors, economists, statisticians, anthropologists, mathematicians and geographers (among others!) to comprehend the interconnections of human behavior, disease transmission, government interventions, global transport and trade, and the production and distribution of vaccines and treatments.

Evolutionary biology is another field that has been crucial for making sense of the COVID-19 pandemic. Examples of evolutionary biology’s importance are many, including identifying selective pressures that lead to the rise of new variants of concern, understanding human responses to disease in relation to past evolutionary pressures from other infectious diseases, and investigating the breadth of hosts that coronaviruses infect and the ecological context of their spillover among hosts. In many cases, these evolutionary perspectives are also crucial to mitigation efforts. For example, phylogenetic approaches can reveal the origins of a new human pathogen from other species, helping guide surveillance efforts in wildlife or domesticated animals, while also revealing transmission pathways among human populations. In the early months of the pandemic, for example, many of us spent hours on NextStrain (https://nextstrain.org/) examining the most up-to-date phylogenies of SARS-CoV-2 to help make sense of its global movement.

We can see the breadth of these evolutionary perspectives on COVID-19 in the pages of *Evolution, Medicine, and Public Health (EMPH)*. So far, *EMPH* has published 24 scientific articles that directly addressed the coronavirus pandemic. Some broad perspectives were published early in the pandemic and helped set the stage for contributions from evolutionary medicine [5, 6]. The articles have continued to flow into the journal over the past 3 years, continuing to the present [7, 8]. Many of these articles highlight the interdisciplinary and collaborative nature of our discipline, with teams that extend beyond evolutionary biology. We have collected these and other papers into a Virtual Issue on ‘Evolutionary Medicine and the COVID-19 Pandemic.’ Here, I highlight some of these contributions in some broad categories to introduce this new Virtual Issue, with the goal to whet your appetite to check out the full collection.

Several *EMPH* papers on the coronavirus pandemic represent rigorous and innovative applications of evolutionary biology approaches to characterize the evolution of the virus and its hosts. One of these studies investigated the functional relatedness of coronaviruses based on physical–chemical features of the receptor binding domain of the virus and compared the performance of this method to standard phylogenetic approaches [9]. The authors’ approach may prove useful in the surveillance of other zoonotic diseases. Another study considered the host perspective by investigating why dogs appeared to be less susceptible to SARS-CoV-2 than cats [10]. The authors found that cats and people share a greater similarity in the receptor angiotensin-converting enzyme 2. Yet another paper investigated the viral evolution of the spike protein in coronaviruses that infect humans and other animals, revealing the importance of recombination [11].

Other studies have investigated the evolution of the immune system and host defenses in relation to COVID-19 treatment, including studies on cytokine storms [12], fever [13] and host-pathogen conflicts over calcium [14]. Another study considered the ontogeny and senescence of the immune system in explaining the age distribution of mortality [15]. One interesting paper in this category considered the hygiene hypothesis, and specifically, whether colonization with helminths and protozoa reduces adverse responses of the immune system to SARS-CoV-2 infection [16]. This perspective has important global health implications, potentially explaining the reduced mortality of SARS-CoV-2 in low-income settings [17]. A more recent *EMPH* paper used a mathematical model to investigate how long-term infection in immunocompromised hosts influences SARS-CoV-2 evolution, thus identifying conditions under which immune escape variants are more likely to evolve [18]. It turns out that fitness valleys are important in this context.

Many other studies in *EMPH* have considered the social context of SARS-CoV-2, often with rich interdisciplinary perspectives. One review used perspectives from evolutionary behavioral sciences to understand pandemic-related behavior, specifically aimed at behavioral changes needed to reduce disease transmission during outbreaks [19]. Another interdisciplinary team applied an evolutionary modeling approach to investigate intervention strategies, with an approach that merged perspectives from epidemiology, evolutionary optimization and economics to target transmission at a level that can be managed by healthcare systems [20]. This research gives new perspectives on adaptive intervention strategies and highlights the importance of implementing mitigation strategies early in disease outbreaks. Finaly, in a paper I co-authored with Alma Solis, we developed the framework of ‘One Health disparities’ and applied it to understand health disparities in the coronavirus pandemic, focusing on the importance of exposure, susceptibility and disease severity at the human–animal interface and within human contact networks [21].

A number of these papers are useful in teaching, both as readings and for slides or examples in lectures. One appealing aspect of *EMPH* is its open-access publishing model, thus giving students access to the papers without paywalls or constraints on article use. In my Evolutionary Medicine and Global Health course at Duke University, for example, Crespi’s ‘Evolutionary medical insights into the SARS-CoV-2 pandemic’ [5] has provided valuable perspective on the spillover of viruses from bats and characteristics of these infections in humans given their long-term evolution in bats. Likewise, my students have engaged with Parker *et al.*’s paper on the connection between missing helminths and SARS-CoV-2 [16], including its rich discussion of what ‘hygiene’ really means in relation to the hygiene hypothesis, with an important distinction between personal hygiene and systems hygiene. It is systems hygiene—such as municipal services that provide clean water and safe processing of fecal waste—that has the largest impact on eliminating helminths and other organisms that modulate immune responses in high-income countries. Finally, I have regularly assigned my students the paper I co-authored on One Health disparities [21], which engages students in a broad set of mechanisms that generate health disparities. Looking through the collection of papers in the Virtual Issue, I see many others that I will include in my teaching in the future, and in my scholarly writing.

I hope this overview of some papers will encourage you to peruse this impressive set of contributions to the journal to learn more about the interdisciplinary breadth of evolutionary perspectives on SARS-CoV-2, its expression in humans as COVID-19, and its transmission within and across species. These articles are valuable in scholarship and teaching and will hopefully entice others to contribute new disciplinary (or interdisciplinary) perspectives on the pandemic, its outcomes, and its eventual control. As a virtual issue, new papers will be added over time. Please submit your papers to *EMPH* and join the collection!

## Notes

Editorial for *EMPH* to accompany the collection of articles, ‘Evolutionary Medicine and the COVID-19 Pandemic’:
